# Toward an epistemology of nano-technosciences: Probing technoscience from a historical perspective: on today’s surprising prevalence and relevance of Francis Bacon

**DOI:** 10.1007/s10202-011-0104-z

**Published:** 2011-12-03

**Authors:** Jan C. Schmidt

**Affiliations:** Unit of Social, Culture and Technology Studies, Darmstadt University of Applied Sciences, Haardtring 100, 64295 Darmstadt, Germany

## Abstract

This paper aims to contribute to the attempts to clarify and classify the vague notion of “technosciences” from a historical perspective. A key question that is raised is as follows: Does Francis Bacon, one of the founding fathers of the modern age, provide a hitherto largely undiscovered programmatic position, which might facilitate a more profound understanding of *technosciences*? The paper argues that nearly everything we need today for an ontologically well-informed epistemology of technoscience can be found in the works of Bacon—this position will be called epistemological *real*-*constructivism.* Rather than realist or constructivist, empiricist or rationalist, Bacon’s position can best be understood as real-constructivist since it challenges modern dichotomies. Reflection upon the contemporary relevance of Bacon could contribute to the expanding and critical discussion on technoscience. In the following I will reconstruct the term “technoscience”. My finding is that at least four different understandings or types of the term “technoscience” co-exist. In a second step, I will analyze and elaborate on Bacon’s epistemological position. I will identify central elements of the four different understandings in Bacon’s work. Finally, I will conclude that the epistemology of technoscience is, indeed, very old—it is the epistemological position put forward by Bacon.

## Résumé

Cet article vise à contribuer aux tentatives de clarifier et de classifier la notion vague de «technosciences» à partir d’une perspective historique. La question clé soulevée est la suivante: Francis Bacon, un des pères fondateurs de la période modern, fournit-il une position pragmatique en grande partie non découverte qui pourrait faciliter une compréhension plus approfondie des technosciences? L’article présente l’hypothèse que pratiquement tout ce dont nous avons besoin aujourd’hui pour épistémologie ontologiquement bien informée de la technoscience peut être trouvé dans les travaux de Bacon. Cette position sera appelée le constructivisme épistémologique réel. Plutôt que réaliste ou constructiviste, empiriste ou rationaliste, la position de Bacon peut être comprise le mieux en tant que constructivisme réel dans la mesure où elle remet en cause les dichotomies modernes. Une réflexion sur la pertinence contemporaine de Bacon pourrait contribuer à l’élargissement et la discussion critique de la technoscience. Ci-dessous, je vais reconstruire le terme «technoscience». L’aboutissement de ma réflexion consiste dans le fait qu’au moins quatre compréhensions ou types du terme «technoscience» coexistent. Dans un second temps, je vais analyser et élaborer la position épistémologique de Bacon. Enfin, je vais conclure que l’épistémologie est, en effet, bien ancienne—c’est la position épistémologique mise en avant par Bacon.

## Abstract (de)

Dieser Aufsatz möchte zu einer Klärung des Begriffs „technoscience“ beitragen. Dazu wird eine historische Perspektive gewählt. Zentrale Frage ist: Könnte nicht einer der Väter des Projekts der Moderne, Francis Bacon, einen grundlegenden Ansatz liefern, um den Begriff „technoscience“ differenziert verständlich zu machen? Dieser Aufsatz möchte zeigen, dass alles, was wir für eine ontologisch wohlinformierte Erkenntnistheorie der „technosciences“ benötigen, sich im Kern in den Arbeiten Bacons im frühen 17. Jahrhundert findet. Bacons Position könnte als epistemologischer Real-Konstruktivismus oder Real-Konstruktionismus bezeichnet werden. Insofern er moderne Dichotomien hinterfragt, passen die klassischen Kategorien—Realismus oder Konstruktivismus, Empirismus oder Rationalismus—nicht mehr. Im Folgenden soll der Begriff „technoscience“ rekonstruiert werden. Es wird gezeigt, dass mindestens vier unterschiedliche Verständnisweisen koexistieren. In einem zweiten Schritt wird Bacons erkenntnistheoretische Position untersucht. Vier zentrale Aspekte können herausgestellt werden. Schließlich sollen diese beiden Gedankengänge zusammengeführt und dargelegt werden, dass Bacons Erkenntnis- und Wissenschaftsprogramm heutzutage weit verbreitet ist. Das heute so scheinbar aktuelle Programm ist damit alles andere als neu; es ist jenes Programm, das zu Beginn der Moderne von Bacon aufs Gleis gesetzt wurde.

## Introduction

Bacon is back—perhaps he has never been away! The physicist Michio Kaku sees late-modern societies “on the cusp of an epoch-making transition, from being passive observers of Nature to being active choreographers. The age of discovery in science is coming to a close, opening up an age of mastery” (Kaku 1998, 16f). The program of intervening, manipulating, constructing, and creating is central to technoscience. Technosciences—such as recent converging technologies (CTs): the synergistic combination of *n*anotechnology, *b*iotechnology, *i*nformation technology, and *c*ognitive science (NBIC)—aim to enable a fundamental constructing and creating of the world atom-by-atom.^1^ In NBIC both technological reductionism and technological constructivism are unified (Schmidt 2004). A technoscience-based “improvement of human performance” is considered desirable as well as feasible (Roco and Bainbridge 2002).

Ian Hacking’s emphatic call for a *Back*-*to*-*Bacon movement* has obviously turned into reality (Hacking 1983).^2^ Today’s technoscience can be considered as a new tip of the iceberg of the Baconian project of modern science and the modern age in general.^3^ Nothing is new and unknown—except some side effects. Nearly everything can be found in Bacon’s work (cp. Krohn 1987). The seeds of technoscience trace back to the origins of the project of the modern age—that is my thesis. To be more provocative, we can draw a line from Bacon to present-day technosciences.^4^

Bacon’s implicit omnipresence today is, indeed, rather surprising. In the 1980s and 1990s, philosophers and sociologists diagnosed an “end of the Baconian age” (Böhme 1993). Scientific and technological progress can no longer be equated with societal and human progress. Environmental problems emerged, revealing the ambivalence of science-based technology: silent spring, acid rain, the hole in the ozone layer, and global change problems. Severe accidents—occasionally labeled “normal catastrophes”—have occurred: Harrisburg, Chernobyl, Challenger, Bhopal, Sandoz, Exxon Valdez. Even earlier, in the early 1970s, the Club of Rome released a first study on *Limits to Growth* and challenged the Western way of life. Hans Jonas argued for a heuristic of fear, a prevention principle, and a new kind of imperative of responsibility (Jonas 1984). In the 1980s, Bacon’s worldview of the technoscientific power to change the world and his *political metaphysics* were identified as *the* historical origin and source of these problems. Bacon became the crystallization point and the addressee of severe critique for the various side effects of science, enlightenment and modernity in general: as *Baconianism*.^5^ Positively and negatively, Bacon’s impact on modern sciences (and the modern age) is documented by the extensive history of Bacon’s reception. Kant, most notably, ascribed the “revolution in ways of thinking” to none other than Bacon in the preface to his *Critique of Pure Reason* (Kant 1989). In Kant’s famous and militaristic words, Bacon had opened up “the highway of science” (in German: Heeresweg der Wissenschaft).

The idea of this paper is to show that reviewing Bacon might serve to foster and substantiate the technoscience discourse. Bacon’s implicit prevalence and relevance in the world of today has, it would appear, largely escaped notice. The provocative suggestion made by this paper is that a little more “materialism” would be very helpful to epistemology, to cope not only with the taciturnity of nature, but also with the harshness of day-to-day reality: technology, economy, society, life situations, working worlds, technological consequences (cp. Feenberg 2002; Frodeman et al. 2010; Ihde 1991). The objective is to argue toward a political philosophy of technoscience (cp. Rouse 1987). Referring to ideas and arguments expressed in the works of contemporary scholars,^6^ I ask: Does Francis Bacon provide a hitherto largely undiscovered programmatic position which might facilitate a more profound understanding of *technosciences* and technoscientific politics*,* in particular of nano-technoscience (cp. Nordmann 2008; Hackett et al. 2008, 26)?

In the following (2), I will reconstruct the term “technoscience” and some lines of the debate on technoscience in order to provide a reference frame for the further investigation. My finding is that at least four different understandings or types of the term “technoscience” are widespread. (3) In a second step, I will analyze and elaborate on Bacon’s epistemological position. I will identify central elements of the four different understandings in Bacon’s work. (4) I will then conclude that Bacon’s program is prevalent in the technoscience discourse: An epistemology of technoscience is, indeed, very old—it is an epistemology put forward by Bacon.

## Technoscience

Besides Gilbert Hottois (1984) and Bruno Latour (1987), Donna Haraway forged the term “technoscience”:^7^ “The world-building alliances of humans and nonhumans in technoscience shape subjects and objects, subjectivity and objectivity, action and passion, inside and outside in ways that enfeeble other modes of speaking about science and technology. In short, technoscience is about worldly, materialized, signifying and significant power” (Haraway 2003, 43).

That is, of course, not a definition. What “technosciences” really are, is hard to tell. The term is incredibly complex (Weber 2003; Nordmann 2005, 2008). As yet, “technoscience” remains an unspecified umbrella term. The lack of clarity, however, is not regarded as a sign of insufficient effort: The complex term “technoscience” seems to mirror an inescapable, complex technoscientific world. That is why the term is considered to elude any attempt at finding a clear definition—a perfect negative dialectic. This non-determinability seems to fit very well with the intentions of those who are engaged in the discourse. Their point of departure is the perception that traditional boundaries, well-established categories, and presupposed dichotomies are becoming blurred *or* have always been blurred, e.g., the boundaries between science, technology and society, between natural and engineering/technical sciences, between biology and technical systems, between theory and practice, between nature and culture, between the given and the fabricated, between autonomy and algorithmicity, between eternal facts and human-made values, between science and politics, etc.^8^

Strikingly, the technoscience discourse evokes major topics widely discussed under the label of “deconstructivism” and, more provocatively, of “postmodernism” in the 1980s. At that time, the hot topics were about the blurring or dissolution of the boundaries considered as constitutive cornerstones for the self-understanding of the modern age. Accordingly, the term “technoscience” extends the postmodernist discourse and brings a fresh flair of postmodernism to the fortress of science and technology. Granted, it might be less provocative to speak about late, reflexive or second modernity and to omit the term “post”; even so, this does not alter the actual content.

Whatever the reasons for the lack of clarity surrounding the term “technoscience”, we are witnessing an ongoing debate on the essential content.^9^ Most significant is the question of whether or not we can draw a line between sciences and technosciences, or between technoscience and engineering sciences. In other words, does an epochal break and historical transformation from science to technoscience take place? Or, in contrast, has science always been a kind of technoscience, implying that there is no historical shift? In the latter case, we can deconstruct and criticize the oversimplified and mystified self-understanding of science of being pure and value-free that has been supported by scientists and philosophers of science for various reasons. Both arguments remain somewhat vague on what can be regarded as criteria for the diagnosis of a transformation or not: Is there a *differentia specifica?*

In addition, there is still vagueness regarding the main area to which “technoscience” should be assigned. Does the term “technoscience” refer to the discourse *or* to reality? In other words, is the discourse on technoscience merely a discourse on the way of seeing, perceiving and talking about present-day science and technology—a discourse on the discourse and on the rhetoric? Is “technoscience”, then, to be considered a term of reflection? Or, in contrast, does “technoscience” refer to technoscientific practice, to the technoscientific objects, knowledge types, and methods?^10^

These questions are far too big to be addressed here and, also, they are far too fundamental to allow a simple straight-forward answer. What I will attempt to do is reduce the heterogeneity of “technoscience”. My aim is to develop a reference frame that will enable the technoscience discourse to be related to Bacon’s epistemology, also in order to provide a critique of the recent predominance of Bacon’s view and the related practices. I will show that “technoscience” has a plurality of meanings without a unifying semantic core. I will also refer to Alfred Nordmann’s list of criteria of “technosciences” (Nordmann 2005, 2008).^11^ Methodologically, I will use distinctions that are most common in (traditional) philosophy of science. At least four heuristically distinguishable, but interlaced understandings are on the table. Those who put forward the notion of “technoscience” do not need to subscribe to all four understandings or all four types of technoscience thesis; selecting one is sufficient.

### Motives, interests, purposes, and power

Technoscience is not regarded as a value-free enterprise that is separated from society. On the contrary, technoscience is governed by societal and economic interests, purposes and goals. Technoscientific knowledge production carries a functionalist (finalist, teleological, strategic, and instrumentalist) dimension. It aims to provide knowledge not as an end-in-itself but as a means: technoscientific knowledge as power to change the world. According to Donna Haraway, “technoscience is about […] significant power.” (Haraway 2003, 43) Knowledge is developed from a technical perspective, from the context of application and implementation, far beyond the traditional demand-pull view.^12^ In addition to both—the context of discovery and the context of justification—the context of application—knowledge generated in “broader, transdisciplinary social and economic contexts”—turns out to be central to technoscience (Gibbons et al. 1994, 4): Mode II knowledge production is nothing but technoscience. Neither scientists nor philosophers can pretend that this instrumentalist type of knowledge is pure and value-free.^13^ The internalist perspective on science on the one hand and the externalist (society, economy) perspective on the other hand are interlaced.^14^ Technoscientific knowledge is assumed to be, in the long run, an adequate instrument to obtain a competitive advantage in the global market, to ensure growth and wealth, and to solve societal problems.^15^ Insofar as interests are the starting point of technoscience, the culturally well-established dichotomy between facts and values is blurred at the very beginning of technoscientific practice. The technoscience thesis also emphasizes the dominance of science in present-day societies. Our societies are knowledge or science societies. A scientification takes place, in particular a scientification of technology and of society in general. A good example is nano-technoscience. Nano-technoscience is, on the one hand, based on cutting-edge natural sciences. On the other hand, its motive is “to improve human performance. [… Nano-technoscience as the core of] converging technologies could achieve a tremendous improvement in human abilities, societal outcomes, the nation’s productivity, and the quality of life—a turning point in the evolution of human society” (Roco and Bainbridge 2002).

### Method, practice, process, and action

Technosciences—and modern sciences—depend heavily on instrumentation and experimentation, on intervention and construction. The technical basis has been stressed by the *New Experimentalism* and the older *Methodological Constructivism*: “We observe objects or events with instruments. The things that are seen in twentieth-century science can seldom be observed by the unaided human senses.” (Hacking 1983, 168) Without intervening, shaping and manipulating, a scientific methodology does not exist. Considering experimentation and intervention means framing science or technoscience from an action-theoretical perspective: as process and practice, including various actors and actants in different or “converging” epistemic cultures (Knorr Cetina 1999; Kastenhofer 2007). Science makes and fabricates knowledge, science creates and constructs facts. Bruno Latour uses “the word ‘technoscience’ from now on, to describe all the elements tied to the scientific contents no matter how dirty, unexpected or foreign they seem […].” The focus is on the “activity of making science and not the definition given by scientists or philosophers of what science consists of” (Latour 1987, 174). According to actor-network theory and “relational materialism”, technoscientific knowledge appears to be socially and technically constructed. The context of discovery, construction and creation—in contrast to the traditionally highly esteemed context of justification with respect to propositions, laws, and theories—is in the focus. This focus is adopted by the ethnographic researchers who claim to open the (Pandora’s) black box of science or technoscience.^16^ The more in detail we look at the sciences or technosciences, the greater the heterogeneity becomes—revealing a disunity (Galison and Stump 1996; Knorr Cetina 1999, 2). Non-reducible non-knowledge is most prevalent (Wehling 2006). The age of *big narratives* with mono-causal and unification-oriented explanations appears to be over. Technoscientific action seems to be much more complex and cannot be decontextualized. Nano-technoscience, for example, is highly dependent upon instruments and experiments—and it also generates instruments. A breakthrough did not occur until the 1980s with the advent of the Scanning Tunneling and Atomic Force Microscope. The same holds for computation and simulation technologies underlying nearly all branches of technosciences.

### Objectivity, evidence, and truth

It seems to be a common view of those who are engaged in the technoscience debate that the theoretical justification and empirical evidence of scientific theories is—if it exists at all—a point of minor importance. Theories do not guarantee objectivity, since the thesis of underdetermination holds:^17^ theories are underdetermined by empirical data. For this reason the technoscience proponents raise concerns about classic notions of objectivity, including a theory-centered approach to truth. The classic position of representational epistemic realism is challenged and largely rejected. Although social and cognitive constructivism continues to attract interest by the advocates of “technosciences”, alternative positions such as action-oriented intervening realism (Hacking 1983) or instrumental realism (Ihde 1991) are highly acknowledged. They provide a materialist grounding and aim to keep in touch with our technoscientific *reality*—a position that is not very far from Dewey’s pragmatism. In addition, actor-network theory conveys implicitly realist assumptions to some extent (Sismondo 2005, 72). Bruno Latour, for instance, argues that a present-day constructivism should also focus on the construction of things and material forms. “A little bit of constructivism takes you far away from realism; a complete constructivism brings you back to it.” (Latour 1990, 71) And Donna Haraway underlines in a provocative way: “To be a construct does NOT mean to be unreal or made up; quite the opposite” (Haraway 2003, 46). The question “Construction of What?” (Hacking 1999) therefore includes the construction of material things. An epistemological *materialist real*-*constructivism* seems to underlie the technoscience discourse.^18^ These explicit commitments to a realist stance—surprisingly also partly present under the big umbrella of constructivism—show that it is not necessary to abandon any kind of objectivity. However, objectivity is not related to explanations based on a reliable theory; the deductive-nomological model of explanation is far too theoretical and far too narrow. On the contrary, objectivity in the realm of technoscience is given via the constructed new phenomenon, the built thing, or the produced object itself. In other words: evidence of knowledge emerges immediately from objects, things, and works: as the truth of things. The mere existence of the object represents the truth. Alfred Nordmann discerns a “collapse of distance between representation and its objects” (Nordmann 2006). Davis Baird forges the term “thing knowledge”, contrasting it with theoretical knowledge (Baird 2004). Truth is constructed with, by and within the objects; it is demonstrated by capabilities to change what is given. To paraphrase Hacking: If you can construct it, it’s true. According to Hacking, construction should be considered as the central statement of “realism”. Facts *and* artifacts, reality *and* constructivity, theoretical, *and* technical, as well as truth *and* object existence, knowledge *and* power are interlaced.^19^ The well-established dichotomy between theory and practice is blurred—it probably never existed and was only a powerful self-stylization. Nano-technoscience, for instance, manifests its truth in the molecular plot of the letters “IBM”. In consequence, scientific truth is not verified by a new theoretical concept of the nanocosm or a theoretical explanation, but by the created (new) nanoobject itself. In this kind of validation, images also play a major role in producing and showing the evidence and objectivity; the objects themselves are mostly invisible.^20^

### Ontology and objects

The question concerning the ontology of the technoscientific objects themselves is the most challenging. What kinds of things are constructed and created in technoscience? Their ontology is not clear at all; for example, are they nature *or* technical systems?^21^ Technical systems appear to be and behave like nature. They have within themselves a principle of motion (i.e., change) and of stationariness (i.e., cessation of change), they grow like life and like an organism; in fact, they *are* also living organism. Traditional boundaries, well-established categories and dichotomies that are still omnipresent in the *life*-*world* are becoming blurred or have always been blurred, e.g., between nature, technology, and culture. Technology is regarded as being (bio-)naturalized and culturized, nature is culturized and technologized, culture is technologized and scientificized, etc.^22^ In order to perceive and argue for hybridization, one has to presuppose an Aristotelian concept of nature as phenomenological nature: the untouched nature that has the principle of motion and of stationariness within itself, and not from humans in the way technology does. Inspired by her ethnographic vision of a Cyborg anthropology, Donna Haraway follows the trails across the culturally established borders and depicts the various entanglements: cyborgs, hybrids, test-tube embryos, transgenetic organism, in vitro fertilization, egg donation, artificial insemination, surrogacy and human cloning. Haraway “attempt[s] to refigure provocatively the border relations among specific humans, other organisms, and machines. […] I call that field site the culture and practice of technoscience” (Haraway 2003, 43). In a similar vein, Karin Knorr Cetina “suggest[s] that this expression [of “molecular” or “cellular machines”] can serve as a master analogy for the ontology of objects in the laboratory: the objects that stand out are not used as organism, they are implemented as machines” (Knorr Cetina 1999, 149). Knorr Cetina explicitly uses the term “ontology” that formerly has been a major signifier of metaphysics and natural philosophy. Traditional questions about nature emerge in the horizon of technoscience, such as: What is Nature in the age of technical production, reproduction and self-production? Time-honored terms, such as Nature, have obviously become blurred and do not provide orientation in the life-world. A hybrid ontology, emerging with the objects, is perceived; Nature is undetermined and, possibly, undeterminable. Nano(bio)technoscience is, again, an excellent example that has inspired the discourse about the ontology of technoscientific objects. Some nanostructures have the capability of self-organization and self-assembly into highly complex systems. In “Engines of Creation”, K. Eric Drexler (1990) presents a highly disputed futurological version of self-organization and bottom-up emergence. Nanobots, aka molecular assemblers, are considered the constituents of soft, or molecular, machinery, including molecular fabrication based on autonomous self-organization.^23^ Nature itself is seen to be productive and constructive; the German idealist Schelling spoke about “Nature as Productivity” and *natura naturans*. This holds for all technonatural hybrid objects. Boundaries are becoming blurred and giving rise to a hybrid ontology: Technical systems are (bio-)naturalized, whereas conversely (bio-)nature is technologized.

### Conclusion

The list is not exhaustive. But it may provide at least some clarity regarding the term “technoscience”, at the same time highlighting a plurality of meanings. The list will subsequently serve as a reference frame for the further investigation of Bacon’s epistemology. The overarching feature and common denominator of the four types of technoscience is the recognition that well-established categories and presupposed dichotomies are becoming blurred *or* have always been blurred. Whether we can, or should, re-establish the categories is at issue. Ulrich Beck and Christoph Lau, for example, argue in favor of a boundary-setting politics in order to (re-)gain boundaries: Without boundaries, societal life is not possible (Beck and Lau 2004).^24^

To summarize, four different types of technoscience thesis are found in the present debate: motive-/purpose-technoscience, practice-/method-technoscience, truth-/objectivity-technoscience, and ontology-technoscience. Those who promote the term “technoscience” do *not* need to subscribe to *all* four types or understandings simultaneously. Acknowledging the recent prevalence and relevance of technoscience requires that at least one of them be supported.^25^ Not everybody will agree to *all* of the above-mentioned types of technoscience. Underlying political or philosophical convictions will determine which of the four types one might consider as the most important and which of them will simply be viewed as inferences or mere consequences. For instance, one may defend truth-/objectivity-technoscience or ontology-technoscience and, at the same time, consider practice-/method-technoscience as a semantically empty term that lacks justifiable criteria.

## Bacon’s epistemology in recent technoscience

It is remarkable that all four different types of the technoscience thesis elaborated above turn out to be quite old. In fact, the origin of the technoscientific program can be found in the programmatic work of Francis Bacon (cp. Krohn 1987; Whitney 1989; Schäfer 1999). Although most present-day epistemologists and philosophers do not refer explicitly to Bacon—though many historians and sociologists worked on Bacon’s account of modernity—his epistemology in particular deserves more attention (cf. Schmidt 2007, 2011). Nearly everything we need today for a materialist epistemology can be derived from a review of the epistemic program put forward by Bacon—a position I tentatively call epistemological *real*-*constructivism*, a conjunction of realism and constructivism, including self-constructivism. The term real-constructivism is also used by Siltala (1998) and Astington (2000), obviously with different connotations. This position clearly differs from classic cognitive constructivism or recent social constructivism. The classic dichotomy or antagonism between constructivism and realism is sublated (“aufgehoben” in the words of G. F. W. Hegel), that is, preserved *and* eliminated. What is *constructed*, built and produced is, then, also part of *reality.*^26^ To be a construct does not imply to be not real (cp. Haraway 2003, 46).

Bacon’s claim at the beginning of the seventeenth century was not unpresumptuous: not only verbal knowledge, but intervention knowledge; not a cognitive-contemplative interpretation of a given reality (traditional “Nature”), but a constructive altering and shaping of the world—precisely in the same tenor as the famous Feuerbach thesis of Marx (cf. Farrington 1979). Knowledge and power are siblings. With Bacon, the idea of a science-based transformation of a transform*able* world emerges—reaching out in time and accessing the future: Man as *homo faber* makes history; a *regnum hominis* is fostered by a *scientia activa*. Bacon’s stance as presented in his *Novum Organon* (1620; cf. Rees and Wakely 2004)—the main work of the famous, but unfinished *Instauratio Magna* (Great Renewal of the Sciences)—reflects the pathos of an epoch breaker: there the *old* Organon of Aristotle and here a *new*, viz. his own Organon; there the sunken Atlantis of Plato, here his *New* Atlantis (cf. Bacon 1966). Bacon assumes that an experimental philosophy *for* the future is both possible and necessary. Thitherto, however, “progress in the sciences” had been held back “by reverence for antiquity, for the authority of those held to be philosophy’s great men and then by giving their consent to all that” (NO I: Aph. 84). Only the characters of “master and pupil”, “not of discoverer and improver of discoveries” had been brought forth (NO I: 15).^27^ Instead of persisting in self-referential thinking, Bacon refers to active skills, tinkering, knowledge and insights gained by craftsmen, doctors and seamen in their (inter-)action with their environment (“Nature”). Traditionally, technical practice was devalued. It was regarded as theoretically insignificant. By contrast, Bacon believes that making and doing are fundamental, but also worthy and capable of improvement. Therefore, technical capability should be paired with theoretical knowledge; mechanics with physics. Bacon’s aim was to found, foster and facilitate natural sciences and science-based technology. He delineated the new (techno-) sciences as an inseparable combination of “light-bearing” and “fruit-bearing”, understanding and intervening, insight and impact.

It has to be conceded that the type of *epistemology* advocated by Bacon is not one that focuses primarily on the theoretical, linguistic or logical conditions of the possibility of theoretical knowledge. It was therefore not a type of epistemology that attracted the attention of traditional philosophers of science who mainly focus on the law-based propositions and the unification project of theoretical physics.^28^ There were, of course, exceptions, such as the pragmatists and in particular John Dewey, who 300 years after Bacon challenged the philosophers in similar words: “The devaluation of acting, making, and fabricating has been cultivated by the philosophers.” (Dewey 2001/1929, 8) In light of the foregoing, I will now present and discuss a list of four dimensions of Bacon’s materialist concept of knowledge—his *real*-*constructivism*—that, strikingly, coincide with the four understandings of technoscience; we can use the words “science” and “technoscience” interchangeably.

### Interests, motives, and purposes

For Bacon, knowledge is not just an end-in-itself, but above all a means-to-an-end (instrumentalism): *for* further knowledge, *for* deeper truth, *for* better instruments and technical systems, *for* a more efficient control and changing of nature, even *for* a *regnum hominis* (cf. Krohn 1987; Schäfer 1999). According to Bacon, “the true and legitimate end of the sciences is nothing other than to supply human life with new discoveries and resources” (NO I: Aph. 81) that “may, to some degree, subdue and mitigate their needs and miseries.” (NO I: 37) For this new kind of knowledge, Bacon holds that “human knowledge and power come to the same thing” (NO I: Aph. 3). He believes that the new, active science is positively utilizable in its very core and could be beneficial to all. Therefore, “the end […] for this science is not the discovery of arguments but of arts”, in particular of technology, techniques and technical systems (NO I: 29). The different objectives of the old and new science lead, Bacon holds, to “different effects. For *one* aims to beat an opponent in debate; the *other* to bend nature to work” (NO I: 29), the latter in order “to command [the] things” (NO I: Aph. 29). Bacon is not, however, given to non-theoretical tinkering with direct utility, as he saw in Leonardo and the artist-engineers of the Renaissance. He believes they went about their work aimlessly, governed by “hazard”, randomness, and trial-and-error rather than by methods (NO I: Aph. 8). The “fruit-bearing knowledge” which Bacon is aiming at presupposes—initially—to some extent “light-bearing” knowledge, meaning a “discovery of causes” (NO I: Aph. 99). “Causes” in this context are not limited to an understanding in the sense of epistemic realism, but can also be regarded from an instrumentalist perspective. Knowing the causes is necessary in order to learn and advance “the art of discovering” (NO I: Aph. 130), in other words, “to discover something to enable everything else to be rapidly discovered by means of it”: an *ars inveniendi* (NO I: Aph. 129). This *meta*-discovery program—the discovery of the logic of discovery—may be regarded as the core of Bacon’s approach.^29^

To summarize, Baconian real-constructivism does not stylize knowledge as being free from value and interest. Rather, it aims at light-bearing and fruit-bearing utility as a way of changing, shaping, and manipulating given reality and creating (or self-creating) *new* realities. Knowledge is regarded from the purposive perspective of problem-solving and utility, power and politics.

### Method, practice, action and the context of discovery and construction

For Bacon, intervention is not just an end, but also the means for obtaining knowledge. The core of scientific investigations is methodology, i.e., the experiment (NO I, Aph. 119), including the instruments (“instrumenta”; NO I, Aph. 2), and the construction of an artificial reality. Knowledge is attained neither by random trial-and-error or technical tinkering, nor by passive observation or pure thinking, but by a systematic process of experimental production and technical activity: Man “does and understands only as much as he has observed by *work* [in nature …]; beyond this he has neither knowledge nor power” (NO I: 45). Bacon does not wish to “put together a history of nature free and unconstrained (when, that is, it goes its own way and does its own work […]) but much more of nature restrained and vexed, namely when it is forced from its own conditions by human agency, and squeezed and molded” (NO I: 39). The most appropriate practical action for learning about (and from) nature, therefore, seems to be intervention, more specifically: *construction* and *creation*. The experiment is (“ontologically”) appropriate to nature, because nature *per se* tends to be taciturn and guards its secrets inside. A passive observer’s perspective, which was later also criticized in the pragmatist’s tradition by John Dewey (1929), is impossible according to Bacon: Achieving knowledge is always an artifact-based operation on things, accompanied by the construction of things, hence an *act*. Facts are rooted in actions, in *facere* (from Latin for “to make”).^30^

What argument does Bacon provide in favor of the experiment? Bacon’s argumentation is clearly anti-sensualistic: The sense “fails us”; it “deserts” and may “deceive” us (NO I: 33).^31^ Only the experiment can, according to Bacon, provide the answer. It leads away from the observer’s perspective to the actor’s perspective of knowledge: Experimenting is an action and a process. The experiment provides, under artificial conditions, control of the boundary conditions and the construction of phenomena and things. The boundary conditions may be varied to ensure reproducibility (and reproduction) and to construct spatial, temporal and inter-personal invariance. Stability and thereby regularity is established by human action. Consequently, Bacon “set[s] little store by the immediate and peculiar perception of the sense, but carr[ies] the matter to the point where the sense judges only the experiment whereas the experiment judges the thing.” (NO I: 35) Therefore, Bacon is not a sensualistic empiricist—“the sense” is *not* the “measure of things” (NO I: 35).

With that, the methodological foundation has been laid without yet having paved the way to how knowledge is actually produced and how evidence is guaranteed. In fact, the experiment is also central to Bacon’s “inductive method” (NO I: 31): According to the latter, it is possible and necessary to “abstract both notions and axioms from things by a surer and firmer way” (NO I: 31/Aph. 18/19)—induction as experimental abstraction. However, “things” are not to be understood in a naïve-realistic way, for instance as being simply given, which Bacon was accused of doing. The thing itself becomes accessible, or is even constructed as such, as something “new” (=*productive* function) in the experiment. In the 1980s, Andrew Pickering spoke in a similar vein of the microphysical “construction of quarks” (Pickering 1984): Experiment therefore does not involve intervention only, but in particular also construction or creation*.* Bacon tells us we should *not* pass from the singular to the universal in a *single* step. Rather, it is important “to educe axioms successively and step by step” (NO I: 31). Bacon’s type of induction (“exclusion or elimination theory of induction”) includes a certain type of experiment-based falsificationism: It proceeds by ways of “exclusions and rejections” (NO II: Aph. 18). In Bacon’s view, “men are allowed only to proceed by *Negatives* at first” to arrive at secure knowledge “after making every sort of exclusion” (NO II: Aph. 15). So “every contradictory instance wrecks a conjecture” (NO II: Aph. 18).^32^ Hans Heussler refuted John Stuart Mill’s interpretation of Bacon as a logical inductionist as early as in 1889, and argued against giving Bacon a “one-sided empiristically biased label” (Heussler 1889, ii).^33^

To summarize, the methodological kernel of real-constructivism has been articulated: the experiment as mediation between empirical realism and active-experimental constructivism. Reality also includes that which is constructed.

### Objectivity, evidence, and the context of justification

Knowledge is knowledge as long as it is progressing, developing, and advancing. Bacon denies any kind of atemporal, eternal truth. He sees truth as being relative, in terms of the “daughter not of authority but of time” (NO I: Aph. 84). Bacon speaks of “scientific advance” and “progress” in a way that will become typical for the modern era. Science is research!

What, then, is *true* knowledge for Bacon? Truth is, in a *double* sense, *not* without works and machinery. True knowledge is in the *first* place one that is brought forth on works—via technically based experiments—using the method of induction and then presented in tables of laws. Since the artifacts are essentially based on (the laws of) nature, the experimentally produced facts and things are nothing else but nature. *Secondly,* true knowledge is one that manifests itself in works. Works are, Bacon writes, “pledges [and guarantors] of the truth”, they designate materialized objectivity (NO I: Aph. 124): “Truth and utility are […] the very same things, and works themselves are of greater value as pledges of truth than as contributing to the comforts of life.” (NO I: Aph. 124)—Works therefore have a dual purpose for what can be regarded as truth, that is to say, in both the genesis of truth and in the manifestation of truth.

However, not only do works benefit the truth—the reverse is also valid: Truth serves as “new pledges of works” (NO I: Aph. 81). If the works fail, it is often due to “ignorance of causes”, to untrue knowledge (NO I: 45/Aph. 3): Truth *is useful* to the production of a work or machinery; in addition, and more important, truth manifests in works. For Bacon, this is not a circle, but an iterative, self-dynamizing scientific process of work production and truth generation. Works and truth are therefore more than closely related. Their spearheads are pointed at the *phenomenally* given nature, which has to be “conquered”, “bent” and “constrained” (NO I: 29/Aph. 3); “Experience” and “judgment” should be “drawn from […] the very innards of nature” (NO I: 33). Although the commonplace phrase *knowledge is power* that has been ascribed to Bacon is not to be found in any passage of his writings, kindred definitions are indeed present, for example when he writes that “those twin objectives, human *Knowledge* and *Power*, do in fact come together” (NO I: 45) and “human knowledge and power come to the same thing” (NO I: Aph. 3). The *knowledge/power* link is certainly not to be understood in a descriptive sense only, but also in a normative one: Only that which is based on might and power, and facilitates a command over nature should be considered as (valid) *knowledge*. This connection is first expressed as a *negation*: Without knowing anything about nature, that is, if the cause-effect relationships are “not known”, the “effect” will be missed and cannot be predicted—this lacks practical use for human purposes. Put in *positive* terms: “that which in thought [=theoretical knowledge] is equivalent to a cause, is in operation equivalent to a rule” (NO I: Aph. 3).^34^ Vico later provided a celebrated formulation: *Verum et factur convertuntur* (cp. Dupuy 2005).^35^ According to Bacon, man is even assigned the task of “writ[ing] a revelation and true vision of the Creator’s footprints and impressions upon His creatures” (NO I: 45). The religious motives of the modern era are hardly to be underestimated.

To summarize, in terms of evidence and validity, real-constructivism means progressive manifestation of truth *in* (and through) works and machinery, therefore *through* real-constructs. The things represent truth.

### Ontology and objects

The above-described understanding of truth incorporates a specific conception of nature. Bacon repeatedly rejects broad systems of natural philosophical speculations and metaphysics; but even he cannot dispense with a pre-understanding of “nature” (cf. Krohn 1987); a minimal amount of metaphysics is indispensible. Bacon rejects Aristotle’s separation of technical systems or technology from nature—and, therefore, conversely the separation of nature from technology. His critique of the Aristotelian dichotomy represents a major contribution to the foundation of the modern era. For Bacon, it is neither necessary nor possible to outwit nature (and her rest and self-momentum) by the action of craftsmen and man-made technology. Rather, technology is what is *possible* within the framework of nature, whereas nature is described by the (sociomorphism) “law” (“the fundamental and general laws constitute the forms” of the bodies; NO II: 5, cp. “axiomatum”, NO I: 99)—and, therefore, not by specific (outer) appearances of our life-world or our senses. Nature is regarded as governed by mathematical laws. Thus, Bacon draws technology under the wide umbrella of (nomological) nature and, in this sense, “naturalizes technology”. Technology is nothing but (possible) nature, governed by mathematical laws; hence, it is impossible that technology is not in accordance with nature (cf. Krohn 1987). Therefore, man—in order to build technical systems—must be “servant and interpreter” of nature; he has to achieve knowledge about nature and has to obey nature (NO I: 45/Aph. 1). By phrasing *natura parendo vincitur* (nature, to be commanded, must be obeyed!), Bacon highlights “parendo” first and then “vincitur”. However, the kind of nature that Bacon wishes to subsequently “vanquish” and “command”, in order to reveal her secrets and shape it, differs from the one he “serves” (“parendo”). The nature to be commanded by means of technical instruments and tools is the type of nature which confronts man as an alien force in his day-to-day life: phenomenal nature, including, for example, the pest. In summary, (a) Bacon extends the understanding of nature to all branches of reality, in particular to technology. In this sense, he is a precursor for a *naturalization of technology*. (b) On the other hand, nature has to be vanquished and shaped by means of technology; so we also find in the work of Bacon the initial point for a *technologization of nature*. To foster the development of science-based technology, Bacon presupposes, entirely in accord with modern physics and late-modern technology, that a lawful “union [=unity] of nature” as *the* “foundation for the constitution of sciences” can “start to set up the sciences [=the project of modern sciences]” (NO II: Aph. 27).^36^

We also find a deeper elaboration on ontology of nature and (nature-appropriate) methods/instrumental techniques in Bacon’s work. The understanding of nature advocated by Bacon can be described as a *reductionism to basic properties or to the tiniest parts* (NO II: Aph. 8), which Bacon assumes to be (ontologically) inherent in nature. “For seeing that every natural action is carried out by things infinitely small, or at least too small to strike the sense, no one can hope to govern or change nature until he has duly comprehended and observed them” (NO II: Aph. 6). For achieving this goal of knowledge, Bacon needs to advance human access to nature: We should proceed from the “entrance hall” to the “inner” of nature. “For as yet we are but lingering in the outer courts of nature, nor are we preparing ourselves a way into her inner chambers. Yet no one can endow a given body with a new nature, or […] transmute it into a new body, unless he has attained a competent knowledge of the body so to be altered or transformed.” (NO II: Aph. 7) To accomplish this, “we shall be led [reduced] to real particles, such as really exist. […] the nearer it approaches to simple natures, the easier and plainer will everything become; the business being transferred from the complicated to the simple; from the incommensurable to the commensurable; from surds to rational quantities; from the infinite and vague to the finite and certain; as in the case of the letters of the alphabet and the notes of music.” (NO II: Aph. 8)

Interestingly, all this does not, however, exclude the self-activity of nature. “Nature” is capable of making something and acting “from within” (NO I: Aph. 4). Bacon assumes a kind of productive self-creativity within (nomological, form-based) nature and acknowledges the emergence of new properties and qualitative aspects in the overall (form) transformation process of matter—in agreement with Aristotle and in dissent with the mathematical laws of mechanics. In Bacon’s line of argumentation, it would be just one tiny step forward to argue in favor of the feasibility of technology-based creativity and self-assembly, as we find in advanced nano-technoscience (Drexler 1990).

In summary, from the perspective of natural philosophy, Baconian real-constructivism claims that nature is nature insofar as it is governed by laws; nature is nothing but mathematical law. On the other hand, there is a type of nature that is phenomenologically given. Technological artifacts and technical systems are not unnatural or beyond nature; they are also, and in particular, natural reality insofar as they are constructed. Furthermore, production is apparent in nature itself: Nature is capable of constructing something.

It is remarkable that the four types of technoscience—remembering, of course, that the term “technoscience” was coined subsequently, in the 1970s—can be found in Bacon’s materialist epistemology: as epistemological real-constructivism. The main ideas behind technoscience, in particular the real-constructivist program, trace back to the origin of modern science in the early seventeenth century.

## Conclusion and prospects

“Clearly,” Hans Achterhuis states, “Bacon’s observation about the transforming impact of technology, made at the beginning of the seventeenth century, is as topical as ever.” (Achterhuis 2001, 2) From the programmatic perspective, certainly, there is no epochal break. Almost everything can be found in the works of Bacon.

The objective of this paper was to underline the relevance and prevalence of Bacon in today’s technoscience by identifying four common dimensions of the discourse. Four different types of technoscience (thesis) are at the bottom of the recent discussion: purpose-technoscience, method-technoscience, truth-technoscience, and object-technoscience. Bacon was, indeed, a forerunner of a real-constructivist materialist epistemology. In spite of Kant’s clear reference to Bacon in the opening to the second edition of the *Critique of Pure Reason* (Kant 1989; sadly, Kant has hardly been interpreted from a materialist point of view), Bacon’s epistemology has been ignored and neglected by sociologists and philosophers of science over the last few decades, although the reality and relevance of Bacon’s epistemology today can hardly be denied and underestimated. Bacon has merely been read from the science policy angle—advocating an externalist perspective on science, not as an epistemologist. Today’s widespread (*implicit!*) epistemological Baconianism poses challenges to philosophers and social scientists alike—and to late-modern societies in general. To meet these challenges, it was an excellent move by Gilbert Hottois (1984), Donna Haraway (1995), Bruno Latour (1987), Don Ihde (2002), Jutta Weber (2003), and Alfred Nordmann (2005) to forge a new umbrella term: “Technoscience” has been on the table since the late 1970s and extensively discussed since the 1990s. For the present-day expanding discourse on technoscience, a (re-)consideration of Bacon’s real-constructivism as well as of the history of technoscientific epistemology might be fruitful.^37^ The societal *future* of knowledge will (and should) not be determined without reflection on its *origin*—in order to strengthen or, at least, to preserve our critical faculties and powers of discernment. A discourse on Bacon could make a contribution in this respect: “For any kind of critique of society a critique of knowledge is indispensable, and vice versa.” (Adorno 1969, 158)

Therefore, reflecting upon the contemporary relevance and prevalence of Bacon means creating a foundation for a critique. Only by considering the underlying materialist epistemology of real-constructivism will we perhaps be able (a) to develop a *critical*-materialist account of recent Baconianism and (b) to provide alternatives to the predominance of materialist approaches and the commanding theory of knowledge. This is a further task. It may turn out that we are not inescapably doomed to Bacon’s real-constructivism and the power-based epistemology of technoscience (Böhme and Manzei 2003). However, this is another story that needs further clarification and a new programmatic effort.

## Footnotes

1 Either directly or by harnessing autonomous self-organizing and self-producing processes, molecular (soft!) machines and engines of creation.

2 The Bacon Renaissance is supported by the so-called *New Experimentalism* and the philosophical (re-)discovery of things, material entities, objects. Until the 1980s, philosophers disregarded or, at least, devaluated engineering and technical sciences—only in the materialist and pragmatist tradition do we find work on Bacon. Particularly outstanding is John Dewey’s approach and his concerns about the predominance of idealist-dichotomist epistemologies. More recently Ian Hacking aimed to “initiate a Back-to-Bacon movement” with his book *Representing and Intervening* (Hacking 1983, 150): “Philosophers of science constantly discuss theories and representation of reality, but say almost nothing about experiment, technology, or the use of knowledge to alter the world” (ibid, 149). Bacon advocates a view of nature and of science that Hacking describes as follows: Bacon “taught that not only must we observe nature in the raw, but that we must also ‘twist the lion’s tail’, that is, manipulate our world in order to learn its secrets” (ibid, 149). Hacking elaborates on what he calls the “Baconian topics”. However, Hacking does not distance himself in any way from the big intervention approach of modern science. We do not find any critique in Hacking’s work. In line with Hacking’s *entity realism* is the *instrumental realism* of Don Ihde, who complained that “Bacon has often been overlooked” (Ihde 1991, 63). Much earlier, in the 1970s, the Starnberg Group (C.F.v. Weizsäcker and others) developed the finalization theory in order to underline the external orientation of science—also with reference to Bacon (Böhme et al. 1983).

3 Also encompassing reflexive modernity (which, certainly, is not always a *reflective* modernity).

4 There is a continuous history of Bacon’s program, visions and ideas (no epochal break with regard to the program). Arguably, Bacon’s ideas might in former times have been regarded as merely programmatic visions that were only partly fulfilled—e.g., the steam engine is not Baconian, it was a result of technical non-scientific craft-knowledge, whereas today his visions seem to have been realized to the full extent.

5 The general ambivalence of the Baconian project of modernity was, in fact, already questioned a few decades ago by Max Horkheimer and Theodor W. Adorno in their *Dialectics of Enlightenment* (Horkheimer and Adorno 1972). Concerns regarding Bacon’s worldview were raised particularly in the 1970s and 1980s; critics accused Bacon of advocating a commanding-conquering view of nature, an instrumentalist concept of knowledge, and a naive linear-optimist model of science-based technological progress as *the* driving force for societal-human progress.

6 Hacking (1983), Latour (1987, 1990), Rouse (1987), Cozzens and Gieryn (1990), Haraway (1991), Bono (1995), Feenberg (2002), Radder (2003), Ihde and Selinger (2003), Nordmann (2005, 2006, 2008), Smith and Schmidt (2007), and Zittel et al. (2008).

7 In addition, Ihde (2003), Weber (2003), Nordmann (2005, 2008) and others advocate the term “technoscience” to describe the historical transformation in the “culture of science”.

8 For instance, the STS scholar Sergio Sismondo regards “technoscience” as a concept that underlines that “scientists and engineers are separated only by traditional boundaries” (Sismondo 2005, 59).

9 However, the indetermination of this term seems to be part of the structure, character and politics of technoscience itself: we lack an adequate terminology.

10 Another point might be added: How are these two aspects interrelated: does the rhetoric determine the future state of science and society? A further question is what should the discourse achieve—a mere description *or* a normative assessment of present-day and/or future technologies?

11 Nordmann identifies various “symptoms for the change of culture from science to technoscience” (Nordmann 2005, 215).

12 According to Gibbons et al. (1994, 5), knowledge is “not developed first and then applied to the context later by a different group of practitioners.”

13 Thus, knowledge is not seen as an end-in-itself, but rather as a combination of means-to-an-end and end-in-itself. Knowledge is to be considered as both truth and utility—the latter as a way of changing, shaping and manipulating *given* reality and constructively creating *new* realities.

14 STS scholars, in line with some Critical Theorists, underscore that facts and artifacts are political (“artifacts have politics”, Winner 1980) and that there is not an essentialist difference between politics and epistemology. According to this view, epistemology is part of the power discourse; in the classic STS terms: “Truth speaks to power” and v.v.

15 Technoscientific knowledge is highly esteemed. For instance, Technology Assessment (TA) might also be framed as a certain type of technoscience.

16 STS scholars find context-dependent and situated practices of science in action. Their laboratory/field studies and their (de)constructivist approach reveal a broad variety of plural, multi-faceted and complex phenomena that resist any unification (Hackett et al. 2008).

17 This is also inherently linked with the classic induction problem and the Duhem-Quine thesis.

18 The position of “real-constructivism” is, as yet, not broadly perceived and discussed.

19 It should be noted that technology and technical systems have never been mere instruments or just the external applications of science; means and ends have never been “ontologically” different.

20 John Ziman’s concerns that the disappearance of traditional academic sciences, including the paradigm of objectivity and fact-based dialog, will have unwanted consequences for democracy itself seem important to be considered here (Ziman 2000). Until now, Ziman argues, science—even if the actual practice might have been different—has served as the paradigmatic ideal of an argumentation-based, power-free discourse.

21 This is the point of departure for the extended debate on the issue of: what does “nature” and “technology” mean in this context?

22 Technology is naturalized: Technology is not—as Aristotle maintained—con-/anti-nature. However, technology is what is possible according to nature. Nature is law-like. For instance, the core of technoscientific systems is governed by nothing but the laws of nature. Nature and the laws of nature have been seen as identical since the very beginning of modern science. Therefore, scientific knowledge is to be considered central to the construction of technical systems.

23 According to Drexler, the self-productivity of nano-assembly could serve as the basis for an engineering revolution in the fabrication of complex systems—leading to a new renaissance and a next industrial revolution of soft machines. “Assemblers will be able to make anything from common materials without labor, replacing smoking factories with systems as clean as forests” (Drexler 1990). Tiny gears, motors, levers, casings, and proteins, genomes, mitochondria, cells, organs will be produced by molecular tools in processes of self-organization.

24 The technoscience thesis does, indeed, advance a strong critique of traditional scientific (theory) realism: In many respects, this kind of realism is revealed to be (nothing but) idealism. The technoscience scholars seem to advocate a kind of materialism, but not a naturalism. In particular, Ian Hacking refers to Bacon and to Marx (and his Feuerbach thesis): Not interpreting, but changing, the world is the goal (Hacking 1983).

25 Cp. Wittgenstein’s family resemblance.

26 Real-constructivism will probably not fit into any established dichotomy schema of epistemology, and in particular will not succumb to a reduction to the cognitive-contemplative. Today, a real-constructivist epistemology could be fundamental to a critical assessment of current technosciences, for example, nano-technoscience. The guiding idea that will not be elaborated on in this paper is: “All questions of epistemology are also questions of social order”, as Latour writes (1999). Reflection on Bacon points to ways of getting back to a critique—and focusing attention on the ambivalence of modernity, its “politics of things” (Latour 1999; cp. Feenberg 2002) or the “technoscientific politics” (Hackett et al. 2008).

27 Bacon extends this critique of the “human authorities” of “antiquity” (NO I: Aph. 84) into an early form of critique of philosophy and ideology—as part of his *epistemology*. In his famous doctrine of “idols” (NO I: Aph. 38ff) Bacon attempts to show how uncertain notions, false judgments and circular systems of thought arise through the reference to authorities.

28 Bacon’s program is, in fact, a theory of science in society, see Cozzens and Gieryn (1990).

29 If *the* source of societal progress lies in the investigation of nature, as Bacon believes, society has an interest and claim in science. An active science requires division of labor and a strong institutionalization—that is to say: suitable general conditions of good scientific practice. “For only then men begin to know their own strength, when instead of countless men doing the same thing, some will be responsible for some things, others for other things.” (NO I: Aph. 113) Bacon delivers a programmatic account of cooperation based on the division of labor, such as was found in early capitalist manufactories, in his utopian narrative “New Atlantis”. “Salomon’s House”—the scientific institution of New Atlantis—is not a subordinate authority of the state “Bensalem”; it is rather an autonomous institution. Scientists are free to decide what is worth knowing. This institutional freedom guarantees light-bearing knowledge and, in consequence, the most fruit-bearing one.

30 This calls the common modern dichotomy between ontology and epistemology into question. Bacon’s and Dewey’s critique was, in principle, confirmed by quantum physics and its measurement process in the early twentieth century.

31 For instance, if the “minuteness of [a body’s] parts” or “its swiftness or slowness” dominates (NO I: 33), the human senses are overtaxed, making a transition from the “incommensurable to the commensurable” infeasible (NO II: Aph. 8). Initially, the idea that it might be possible to refine the senses by means of instruments, for example a microscope, could occur. That, however, would only shift the problem gradually—it is not sufficient.

32 According to Bacon, inductive method means a tentative interplay between exclusatory inductions, experimental constructions and deduction. In this way, broader statements can be obtained successively. By means of this method, science is “driven on, as it were, by a machine”. In the nineteenth century, Bacon’s accounts led some philosophers, such as John Stuart Mill, to discern in them only a contribution to inductive *logic*—not to inductive *methodology*.

33 Heussler’s rationalist’s line of interpretation could be supplemented with an interventionalist’s, or more specifically: a real-constructivist’s one. The inappropriateness of a mere inductivist interpretation is also substantiated by Bacon’s prominent example of the *bees*, in which he contradicts both the Renaissance engineers and pure empiricists *and* the school philosophers and rationalists. Bacon likens experimentalists and empiricists to “ants” who merely “store up and use things”; they are mindlessly just gathering data. Rationalists, on the other hand, are like “spiders” that “spin webs from their own entrails”, e.g., they are spinning empty theories. Bacon’s ideal is the bee, which “takes the middle path”. The bee “collects its material from the flowers of fields and garden, but its special gift is to convert and digest it” (NO I: Aph. 95).

34 The term “rule” might misleadingly suggest a Humean kind of “rule following” in the sense of a nomological succession of events—or a Newtonian trajectory of an oscillating body. That is not what is meant by Bacon; for Bacon, rule signifies an action rule or instruction: Whenever I wish to bring about an effect Y, I must do X. In this respect, Bacon echoes in some degree the view of action theorists, for example Georg Henrik von Wright (1971). Von Wright distinguishes between doing and bringing about. *Doing* represents the specific action of man, viz. to posit causes and to set initial conditions, and thereby to induce the regular succession of events in nature, whereas an intended effect, the *brought about event*, results in *and* via nature, e.g., via trajectories based on Newtonian mechanics. In order to act intentionally *in* nature, knowledge of the mechanism of nature is necessary; thus knowledge of nature becomes central to the knowledge of action and production. *Intervention* and *representation* are mutually dependent.

35 That might have irritated traditional religious lines: nature in the hand of man—*is that not hubris?* On the contrary, Bacon believes: the intervention dimension of knowledge is even motivated and justified from a religious perspective. Bacon speaks of God’s “first fruits of creation” (NO I: 45) and goes on to encourage the “imitation of God’s works” by the action of man: as a *second creation*. “Discoveries are also like new Creations” (NO I: Aph. 129).

36 Bacon also criticizes the Aristotelian Four Causes doctrine. Most prominently, he presents—what was to become characteristic for modernity—a critique of the final cause, which “is far from being beneficial that it actually corrupts the sciences” (NO II: Aph. 2). Finality, according to Bacon, is scientifically non-perceivable; it is just metaphysics. After all, in nature “nothing really exists […] besides individual bodies, carrying out pure, individual acts according to law, yet […] this very law, and the investigation, discovery and explanation of it, is the very foundation […] of knowing as it is of operating” (NO II: Aph. 2). So Bacon already introduces an understanding of laws into the center of the new experimental natural philosophy. The aim is to find “the general and fundamental laws which constitute forms” (NO II: Aph. 5). Admittedly, the notion of form may still sound somewhat Aristotelian; but forms are reinterpreted as underlying laws.

37 Moreover, a specific Baconian real-constructivism might also be found in the diagnoses on the state of current sciences—a point that has to be elaborated on elsewhere, for instance in theses on: *New Production of Knowledge/Mode 2,**post*-*normal* or *post*-*academic science.*
